# Molecular simulation grid

**DOI:** 10.1186/1758-2946-3-S1-O17

**Published:** 2011-04-19

**Authors:** Jens Krüger, Georg Birkenheuer, Dirk Blunk, Sebastian Breuers, André Brinkmann, Gregor Fels, Sandra Gesing, Richard Grunzke, Oliver Kohlbacher, Nico Kruber, Ulrich Lang, Lars Packschies, Ralph Müller-Pfefferkorn, Sonja Herres-Pawlis, Patrick Schäfer, Hans-Günther Schmalz, Thomas Steinke, Klaus-Dieter Warzecha, Martin Wewior

**Affiliations:** 1 Department of Chemistry, University of Paderborn, Germany; 2 PC2, University of Paderborn, Germany; 3 Department of Chemistry, University of Cologne, Germany; 4 Bioinformatics Department, Eberhard-Karls-University of Tübingen, Germany; 5 ZIH, Technical University of Dresden, Germany; 6 RRZ, University of Cologne, Germany; 7 Faculty of Chemistry, University of Dortmund, Germany; 8 Zuse-Institut, Berlin, Germany

## 

MoSGrid is the acronym for Molecular Simulation Grid, a BMBF funded joint research project with the aim to offer grid services for the broad field of molecular simulations in the D-Grid infrastructure. Besides tendering various codes ranging from quantum molecular calculations (e.g. Gaussian, Turbomole) via molecular dynamics (e.g. Gromacs) to docking approaches (e.g. FlexX) for high performance computing, one of the main goals is the integration of metadata annotation for data mining and knowledge generation.

Molecular simulation codes and computational resources are accessed via the MoSGrid portal (http://www.mosgrid.de), which will offer intuitive access to various tools and will support the users with workflows, for an easy import of molecular data, a simple setup and submission of calculations as well as extraction of relevant results. The portal will hide the complexity of the underlying technology by providing a unified user interface making computational chemistry in general more readily available.

MoSGrid’s server-based portal is available as open-access and open-source software. Users are relieved from software installations and do not need to have knowledge about the underlying infrastructure. The portal includes portlets specifically set up for the various simulation programs. Commonly used workflows, simple or complex, can be stored in recipe repositories and are available for every user. Moreover, users can develop, improve, publish, and use workflows for their everyday tasks.
